# Practical Observations on the Knowledge and Cure of Chronic Deafness

**Published:** 1834-04-01

**Authors:** 


*Erfahrungen über die Erkenntniss, und Heilung der langwierigen Schwerhörigkeit.* Von Dr. W. Kramer.—Berlin, 1833.

*Practical Observations on the Knowledge and Cure of Chronic Deafness.* By Dr. W. Kramer.—Berlin, 1833. 8vo. pp. 106.

In the review of Mr. Lawrence's work, in our first number, we ventured to assert that medicine has its debateable ground; that, in the empire of the healing art, as in some disorderly, half-civilized state, there are distant tracts over which the authority of the scientific practitioner seems scarcely established, and where the noisy charlatan (like the moss-trooper of old,) bears sway, in the absence of some more respectable functionary. Now, many judicious persons are disposed to think that all which concerns the art of curing the diseases of the auditory apparatus is at present in this unsatisfactory state, and they are apt to compare aurists to those "juggling fiends" of whom *Macbeth* speaks:

> "That keep the word of promise to our ear,
> And break it to our hope."

The passage, indeed, from which we have quoted, seems especially designed for the reprehension of semi-medical writers, for they really "palter with us in a double sense:" while one eye is directed to the profession, the other, by a kind of authorial strabismus, is turned to the public; and their books, between these two sources of patronage, of course, fall to the ground. This strong and natural prepossession against ear-books must not, however, prevent our readers from listening to Dr. Kramer; for he is a sober and a candid practitioner, narrates his failures as well as his successful cases, and has produced a work worthy of the highest commendation.

In the introduction, Dr. Kramer observes that total blindness is not so great a misfortune as total deafness: he who is deprived of sight can associate freely with his fellow-creatures, and take part in the merriest conversation; while the deaf man distrustfully contracts more and more the circle of his acquaintances, suspiciously watches every motion of the lips of his best friends, and, retiring from cheerful society, in which he cannot participate, sinks deep into gloomy reveries. It might naturally be expected, therefore, that the pathology of the ear should have been studied with as much zeal as that of the eye. But this is far from being the case; for, while ophthalmological books are sufficiently numerous to form a considerable library, the list is short indeed of those writers who have studied diseases of the ear; and still shorter is the catalogue of those who have studied them with advantage.

Itard deserves the first place, for his work entitled "*Traité sur les Maladies de l'Oreille et de l'Audition*," which is a treasure of well-founded observations on the acute and chronic diseases of the organ of hearing; but the latter, unfortunately, have not been treated with so much attention as the former. "Far be it from me," says Dr. Kramer, "to wish by this remark to make the great merits of Itard appear less; but he is certainly in the wrong when he treats of tinnitus aurium as if it were an independent disease, though it is without exception merely a symptom accompanying the most varied forms of diseases of the ear,—when at one time he divides chronic deafness into species, according to the causes which produce morbid changes in the organ of hearing, and at another time, according to these morbid changes themselves; and we must deeply lament that he passes over the treatment of chronic deafness so lightly, since its frequent occurrence, and obstinate continuance, demand the most attentive and circumspect treatment."

Next to Itard, Dr. Deleau deserves especial mention, though unfortunately he wastes his strength in little ephemeral essays, in order that his eagerness for distinction may be gratified by being continually before the public. In every page of his writings he cries up his own great merits, which, when accurately investigated, may be resolved into this one point, that, in treating diseases of the Eustachian tube, he has substituted the air-douche for watery injections; the advantage of which is not so preponderating as he would have us think. All the remaining writers, says our author, have advanced the study of these diseases but little, or not at all; they content themselves, for the most part, with repeating the errors and prejudices of their predecessors, not even noticing well-established truths: thus, for example in the writings of Curtis, Riedel, Beck, and others, we find no mention of Itard and his excellent work.

In treating chronic deafness, it is necessary to observe accurately the distance at which the patient can hear; for without this it is impossible to judge of the intensity of the disease, or of the value of the treatment pursued. This has been hitherto but little done, and hence the small value of the cases published. Perfect deafness is very rare, and is always incurable; it cannot be meant therefore, when we read, as we so often do, that the patient had lost his hearing. It is, of course, a matter of great interest to know to what degree the patient's hearing has suffered in each single case; and for this purpose Itard invented his *acoumètre*, (*Traité, &c.* vol. ii. p. 50,) an instrument to measure the distance at which a given sound is heard. In the whole of his work, however, there is not a single instance in which he made use of this instrument; a circumstance which greatly weakens our opinion of its utility.

Dr. Kramer uses his watch for this purpose: its ticking can be heard by a healthy ear at the distance of twelve or fourteen ells, when all is still around. This is undoubtedly a very convenient instrument, and the only objection to its use consists in the immense variety of strength in the ticking of different watches: if the reader should happen, like ourselves, for example, to have a watch with Geneva works, he will find that none but *Fine-ear* himself could hear its ticking at the distance of twelve ells.

After describing the anatomy of the organ of hearing, Dr. Kramer gives his method of examining, or, as he terms it, catheterizing the Eustachian tube. He uses for this purpose a silver catheter, five inches and a half long, and varying from the thickness of a crow-quill to that of a goose-quill; it is straight, excepting for five lines near its point, where it is bent at an angle of 144°, in order to correspond with the entrance of the Eustachian tube. The catheter is of equal thickness throughout, excepting at the external extremity, where it is broader, in order to admit the pipe of an injection syringe. At this end of the catheter likewise a ring is soldered on, to shew by its situation the position of the point after it has been introduced into the nose. The Eustachian tube is examined in the following manner: The patient being placed on a chair, the operator sitting down before him, takes hold of the catheter immediately above its funnel-like expansion, keeping the concavity of the catheter downwards; and then, bringing the point into the lower nasal meatus, he pushes it quickly, but carefully, into the fauces. This manœuvre must be executed with a delicate and sure hand, partly in order to spare the patient pain, and partly in order to overcome with success all the difficulties which stand in the way of the catheter, from the lateral deviation of the septum narium, and from irregularity in the structure of the nasal muscles, difficulties which cannot be surmounted by rule.

The catheter being now introduced so far as to touch the posterior wall of the fauces, (till when the ring, and consequently the point, are directed downwards,) the handle must be raised, and the point will glide over the roundish prominence of the *hamulus pterygoïdeus*, and, touching the posterior wall of the *velum palati*, will compel the patient to swallow; while this takes place, the operator turns the catheter one quarter round its axis outwards and upwards, and it enters the Eustachian tube. It is now arrested by the cartilaginous ring at the entrance of the Eustachian tube; the most certain sign for an experienced hand that the catheter has been properly introduced. The ring of the instrument is now horizontal, or even a little turned upwards, and the patient experiences no pressure or inconvenience from this little operation. The process is much facilitated if the calibre of the catheter exactly corresponds with the nasal meatus of the patient, so as perfectly to fill it up. The catheter being now properly situated in the entrance of the Eustachian tube, the operator blows into it, either with his breath or with a small pair of bellows; the patient then feels the stream of air pressing against the membrana tympani, and attempting to force a passage outwards; a proof that the Eustachian tube and the cavity of the tympanum are free from any mechanical obstruction. But, if the patient has not this sensation, we may suppose the existence of mucous accumulations, strictures, or adhesions, in these cavities: the method of distinguishing these will be detailed afterwards.

Passing over the rest of the introduction, we come to Dr. Kramer's division of the chronic diseases of the organ of hearing. These he divides into three classes:

1st. Diseases of the external ear, *i. e.* of the external meatus.

2dly. Diseases of the middle ear, *i. e.* of the Eustachian tube, and the cavity of the tympanum.

3dly. Diseases of the inner ear, *i. e.* of the labyrinth.

The first class again is subdivided into

1st. Erysipelatous inflammation of the lining membrane of the external meatus.

2dly. Inflammation of the lining membrane, with a disposition to polypous growths.

3dly. Inflammation of the lining membrane and the subjacent cellular substance.

The following are the symptoms of the erysipelatous inflammation: The patient, if he attends to his feelings, observes an itching, prickling, burning, or tearing sensation in the meatus, which causes him to thrust his finger into it; to this are soon added tinnitus aurium, with oppressions and a sense of stretching and fulness in the meatus; there is even confusion and heaviness of the head, (on *one* side only, if but *one* ear is affected,) which become very troublesome, and, together with the tinnitus aurium, even prevent the unshackled activity of the mind.

It often happens that these symptoms of inflammatory excitement entirely escape the observation of the patient, but the difficulty of hearing is never absent: indeed, it is sometimes so great, that the ticking of a watch placed on the affected ear cannot be perceived. The meatus becomes filled with morbid cerumen, so tough that, when the case has been long neglected, it concretes into dirty-grey calcareous masses, the removal of which causes considerable pain, and even slight hemorrhage. The cure is easily affected by injections of warm soap and water; and, should any ulcers remain after this treatment, they are to be touched with the Tr. Thebaïca, or the Tr. Myrrhæ.

Our author observes, with great justice, that the erysipelatous inflammation of the meatus, with its product, the accumulation of morbid cerumen, are well calculated to give reputation to empirical remedies against difficulty of hearing, —such as oily and spirituous substances dropped into the ear; though their success in these instances is apt to induce the erroneous belief that they are of general efficacy against deafness, whereas they are of service only when the disease just described is uncomplicated, and even then watery injections are better.

The following is a good example of our author's clear and sensible manner of narrating his cases.

"H. v. G., aged fifty-four, complained to me of having been deaf of his left ear for the last six months: in fact, he could not hear my watch, even when placed upon it. He had never had tinnitus aurium, and the right ear was perfectly healthy. I found a great mass of dark-brown tough cerumen in the left meatus, which was perfectly cleared out, as early as the second day, by injections of lukewarm soap and water. An ulcer, with elevated edges, was now to be seen at the bottom of the meatus, near the tympanum, of the size of half a large pea; rather large blood-vessels ran from it to the tympanum. The ulcer was touched with the Tr. Thebaïca simpl., and healed in a few days; when the tympanum also recovered its natural brightness and transparency, and the patient could hear at the normal distance." (P. 25.)

When there is a polypus in the meatus, Dr. Kramer observes, that the symptoms are occasionally mistaken for those of a threatening of apoplexy, and uselessly treated by venesection, &c. In cases of polypus, our author has never seen the membrana tympani penetrated, or the cavity of the tympanum destroyed by caries of the bones; for it would seem that the morbid activity is always directed outwards, and is concentrated in the polypi and their secretion.

The prognosis is almost always unfavourable; for, although the polypi can be removed in several ways, it is impossible to destroy their roots, especially when they are situated on the membrana tympani, so that new ones are certain to spring up. Moreover, when the membrana tympani itself is the seat of the polypi, it suffers alterations in its structure which unfit it for its office as an acoustic instrument. It is better to remove the polypus with the forceps than by the ligature or the knife; but, should no one of these methods be practicable, recourse must be had to caustic.

In the third subdivision, the inflammation extends to the cellular substance beneath the membrane lining the meatus; there is a purulent discharge, and the meatus, particularly in its deeper parts, is reddened, and, its membrane being swollen, the calibre is necessarily contracted. The process of inflammation and destruction sometimes extends to the membrana tympani, the ossicula auditûs, the substance of the os petrosum, and even to the membranes of the brain and the brain itself. The diagnosis is rendered easy by the speculum auris, but the treatment is generally long, and the result is dubious. The treatment should be constitutional as well as local, says Dr. Kramer: he differs in this point, as he observes, from most practitioners; for example, from Saunders and Curtis, who advise, as in discharge from the ears, to begin and end our treatment with astringent remedies, almost without exception. When the inflammation is considerable, a number of leeches are to be applied round the ear, and repeated, if necessary; but, in such cases, fomentations and vapour-baths are almost always injurious. A very strong tartar-emetic ointment, made with one drachm of the salt to three drachms of fat, should also be rubbed on the mastoid process of the affected side, and the suppuration which arises from it must be kept up for a long time. During the first few days of the suppuration, the discharge from the meatus becomes more copious, but then decreases from week to week, and becomes less sharp, while the inflammatory state of the auditory passage subsides, together with its accompanying symptoms. It is only in the most desperate cases that we are to pass a seton through the back of the neck, cut off the hair, and apply dry friction to the head, as Itard recommends. When the inflammation has been considerably moderated by these means, but the contraction of the meatus still continues, (as it commonly does,) it is time to act directly on the meatus itself. This is done by introducing strips of sponge, the size of which is gradually increased as the passage becomes more and more dilated. The introduction of the sponge must be accomplished with a light hand, otherwise unbearable headach will be the result. When all the advantage has been derived from dilatation that it is capable of affording, but the discharge still continues, then comes the time for the application of astringent remedies, such as the acetate of lead, alum, and the sulphate of zinc: our author prefers the last.

In the second division of his work, Dr. Kramer treats of only three diseases, namely, 1st. Catarrh of the Eustachian tube, and the cavity of the tympanum; 2dly. Contraction of the Eustachian tube; and, 3dly. Adhesion of the sides of the Eustachian tube. The diagnosis of the first of these diseases is generally certain. The catheter is introduced into the Eustachian tube, in the manner already described, and, on blowing through it, the air either does not penetrate the masses of mucus at all, or does so with great difficulty, and with a rattling noise. If this is not sufficient, watery injections may be used; and, if they again do not suffice, a piece of catgut may be passed through till it touches the membrana tympani, thus distinguishing a mucous obstruction from a perfect closure of the tube.

The prognosis is generally favourable. The treatment must be in the first place local, and in the next a general one, intended to amend the mucous and spongy constitution of the patient. In recent cases the first alone is sufficient; and even in old catarrhs the cure is chiefly dependent upon it. The first indication is fulfilled by injections, which have been thrown in by three different methods; namely, through the tympanum, the mastoid process, or the Eustachian tube: but the first two methods are not without difficulties, nay, more, without danger for the integrity of the organ of hearing.

Whether the tympanum has been surgically punctured or been penetrated by suppuration, from its retired position it is impossible to put the point of the syringe exactly on the opening in the membrane. But, if the injection is thrown merely into the external meatus, and that with great force, as Itard recommends, either the opening in the membrane must be considerable if the injection is to have any effect, or the stream of water is much more likely to destroy the membrane entirely, than to force the accumulated mucus through the Eustachian tube into the fauces.

If, in consequence of destruction by caries, there is an opening in the mastoid process, through which injections can be thrown into the cavity of the tympanum, we shall not derive any benefit from this circumstance; for the force of the stream of fluid is so much broken by the cellular septa in the mastoid process, that the intended effect upon the mucus in the cavity of the tympanum will be nullified. As to perforating the mastoid process, in order to inject the fluid, this is not only useless, for the reason just stated, but also dangerous, as Arnemann has ably demonstrated, in an express treatise, entitled "Remarks on the Perforation of the Mastoid Process, &c." (*Bemerkungen über die Durchbohrung des* Processus Mastoideus *in gewissen Fällen der Taubheit.* Gott. 1792.)

We must necessarily omit the rest of Dr. Kramer's very interesting remarks on this particular disease, and content ourselves with giving one of his cases.

"B. E., aged thirty-four, a man of a weakly constitution, slept, during the severe winter of 1829-30, in a cold damp room, where, in the evening, he often found his bed frozen quite hard. From these circumstances, he lost his hearing to such a degree, that he was obliged to give up his situation as servant. In the beginning of May he could no longer hear my watch when placed on his left ear, but he could when it touched the right one. Moreover, he was affected with so violent a tinnitus aurium, that he never had a sound night's rest. Both auditory passages were clear, but the membrana tympani on both sides was dim and opaque. Both Eustachian tubes were obstructed; but an injection (of lukewarm salt and water) made its way through the right one to the tympanum at the very first sitting, and washed out small clots of coagulated blood; after which the patient could hear my watch with his right ear at the distance of two feet. The injection did not penetrate the left Eustachian tube till the third sitting. After the fourth, the tinnitus aurium was entirely gone, and the patient could force the air as far as the tympanum on both sides, and hear the watch at a healthy distance. Two days after this fortunate result had been attained, he suddenly lost his hearing in the right ear, and this occurrence was accompanied by violent pain and a loud tinnitus in it; but these symptoms spontaneously disappeared in a few hours, and his hearing has ever since been perfect." (P. 70.)

When the Eustachian tube is contracted, the cure is to be attempted by dilating it with catgut bougies, the size of which is to be gradually increased; but Dr. Kramer's endeavours have always been fruitless when the stricture has been in that part of the tube which runs in the bony canal. In such cases the perforation of the tympanum is indicated, in order to give access to the atmosphere, and, by thus putting the tympanum on the stretch, to render it capable of transmitting the vibrations of sound to the ossicula auditûs. In spite of the theory, Dr. Kramer has never seen the operation beneficial. The indications for this operation are of rare occurrence, for it is highly improper to perform it merely as a last resource against obstinate deafness.

The diagnosis of adhesion of the sides of the Eustachian tube is exceedingly difficult; as it depends on the operator being unable to find the entrance of the tube: it therefore depends on his consummate dexterity, for an ordinary person may easily fail in finding the aperture. The prognosis is of course unfavourable in the highest degree.

In the last division of his work, the affections of the inner ear, our author treats but of two diseases, irritative and torpid nervous deafness. The latter is to be distinguished from the former by the absence of tinnitus aurium. Although constitutional remedies are not to be neglected, and the health of the patient is of course to be brought into the best possible state; still, says our author, the nervous deafness itself can be cured only by local applications. Now, it is useless to drop remedies into the external passage, as the membrana tympani cuts off the communication with the cavity of the tympanum and the auditory nerve: they must, therefore, be introduced into the Eustachian tube. Gaseous injections alone can be borne; and our author, after experiments with a variety of substances, has found the diluted vapour of acetic ether best adapted to this purpose. He gives several cases in which this method was tried with great advantage; and we must confess it seems rational, as well as ingenious.

Such are the principal points in Dr. Kramer's work; yet we have reluctantly omitted details of considerable importance. In truth, this excellent little treatise is not capable of much abridgment; for to the characteristic merits of German books, depth and fidelity, it adds one which is rarely to be found in them—the most classical brevity.

